# A cross sectional survey of internet use among a highly socially disadvantaged population of tobacco smokers

**DOI:** 10.1186/s13722-019-0168-y

**Published:** 2019-10-15

**Authors:** Sam McCrabb, Laura Twyman, Kerrin Palazzi, Ashleigh Guillaumier, Christine Paul, Billie Bonevski

**Affiliations:** 1 School of Medicine and Public Health, Faculty of Health and Medicine, University of Newcastle, 1 University Drive, Callaghan, NSW 2308 Australia; 2 Cancer Council New South Wales, Woolloomooloo, NSW 2011 Australia; 3 Hunter Medical Research Institute, University of Newcastle, New Lambton, NSW 2305 Australia

**Keywords:** Smoking cessation, Social disadvantage, Internet utilization (mesh term)

## Abstract

**Background:**

Tobacco smoking is highest among population groups which are the most socially disadvantaged. Internet-based smoking cessation programs have been found to be effective, though rates of internet access are not well known in these groups. This study describes the rates of internet use and types of technology used to access the internet by a population of socially disadvantaged smokers. The study also examined relationships between sociodemographic and smoking behaviours with amount of internet use and type of device used.

**Methods:**

A cross-sectional survey of 369 clients (response rate 77%) from two non-government community service organisations in metropolitan New South Wales, Australia was conducted using touchscreen computers. Descriptive statistics and logistic regressions were used to examine results.

**Results:**

Eligible participants ranged from 19 to 88 years old current tobacco users. Over half (58%) of the participants reported weekly or more frequent use of the internet with less than a third (28%) not having any access. The odds of using the internet at least weekly decreased with age and as heaviness of smoking increased (OR = 0.94, p < 0.001; OR = 0.81, p = 0.022, respectively). Odds of internet use were higher as income increased (OR = 2.74, p < 0.001 for individuals earning $201–$400 per week; OR = 2.83, p = 0.006 for individuals earning > $400 per week). Device use differed for age and income.

**Conclusions:**

Internet-based interventions appear to reach the majority of socially disadvantaged populations. It is expected that this reach will continue to grow, making internet-based interventions a potential platform for providing care to low socioeconomic individuals who smoke, however inequalities may be exacerbated for those individual without internet access.

**Implications:**

Internet use among socially disadvantaged tobacco users is moderate (58%). An internet-based smoking cessation intervention for socially disadvantaged tobacco users may be an effective intervention however, older, heavier tobacco users may not benefit as easily due to limited internet access and therefore acknowledging these limitations when developing an intervention can help to acknowledge limitation of intervention reach.

## Background

The prevalence of tobacco smoking is high amongst more socially disadvantaged groups (i.e. the long-term unemployed, homeless, mentally ill, ethnic minorities, prisoners, at-risk-youth, and single parents are some of the groups that collectively can be defined as “disadvantaged individuals”) [1], and is especially true for people with comorbidities such as other substance use and mental health issues in most countries [2–4]. Social disadvantage is related to increased rates of concurrent use of tobacco and cannabis [5–7]. Concurrent daily tobacco and cannabis use has been found to be high (40–78%) as cannabis users often mix their cannabis with tobacco for smoking [8]. Concurrent use has been linked to an increased risk of and higher levels of nicotine dependence, poorer health outcomes and greater difficulty when quitting [8–11].

Delivering effective smoking cessation support to the highest number of smokers in hard to reach groups is a key international priority for tobacco control [12]. Too, the RE-AIM model, which outlines fives aspects related to the impact of public health intervention and can be used to plan or evaluate interventions, highlights that increasing the reach of interventions is essential to address health inequities in society [13]. An internet-based intervention may provide an option for this as internet-based intervention have been found to address the APEASE criteria for designing scalable interventions of Affordability, Practicality, Effectiveness and cost-effectiveness, Acceptability, Safety and Equity [14].

Access to the internet has greatly increased in recent years with home access rates increasing from 83% in 2012–2013 to 86% in 2016–2017 in the Australian general population [15, 16]. By June 2018, there have been an additional 14.5 million internet subscribers in Australia [17]. Smartphone access may increase internet availability further, with a total of 27 million mobile handset subscribers by June 2018 [17]. Differences have been found for device used to access the internet, with the majority of devices used increasing in the 2016–2017 year [15]. However, this data is not available for socially disadvantaged populations. Too, internet access tends to be most prevalent among the more socioeconomically advantaged groups in society, with differences found by education [18, 19], income [18, 19], ethnicity [18], age [19–21], gender [19, 22], and marital status [23]. These factors may too influence internet access among the socially disadvantaged population. Currently there is a dearth of information on the rates of internet use by socially disadvantaged populations. Australian estimates for the rates of household internet access specifically for lowest socioeconomic (SES) households in Australia (calculated by Socio-Economic Indexes for Areas Index) are low, with only approximately one in three households (35%) reported to have internet access. Given this, and it is important more estimates of rates are reported to determine if an internet-based intervention may be applicable for this population group.

A meta-analysis by Boland et al. [24] suggests that websites are effective at increasing smoking cessation among disadvantaged groups (rural Americans smokers, low SES smokers, smokers experiencing a mental illness, African Americans smokers, HIV positive smokers, smokers with a substance use disorder, and Maori smokers) by 37% at 6-months (OR = 1.37, 95% CI 1.01, 1.85, p < 0.05). Previous research suggests that individuals with the following smoking behaviours were more likely to be recruited to an internet-based smoking cessation intervention: smoke at higher rates, smoke within 30 min of waking, had made more quit attempts in the previous year, and had started smoking at a younger age [25]. As such the effectiveness of internet-based interventions may not accurately reflect their impact on the greater population of tobacco smokers and for internet-based interventions to have optimal impact, it is important comorbities and barriers to access of the target population (i.e. concurrent cannabis use, smoking related variables, age, gender, income, education, ethnicity, marital status, and devices used to access the internet) be determined so approaches to address these can be incorporated into an interventions.

Given this, we hypothesis that rates of internet use in this sample would be higher than expected and that devices used to access the internet would vary (i.e. not everyone would own a computer but may be access the internet through other means). This could indicate the potential for an internet-based intervention, deliverable via multiple devices, to offer an affordable and wide reaching method for increasing the provision of smoking cessation care to an at risk population. We also hypothesise that socially disadvantaged people who smoke may be impacted by additional barriers (such as higher rates of tobacco use and cannabis use, and other smoking behaviour variables) which may impact cessation attempts, and the effectiveness of any intervention. For this reason, this study describes the rates of internet use and type of technology used to access the internet by a socially disadvantaged population of smokers. This study also examines relationships between sociodemographic and smoking behaviours with amount of internet used and type of device used. To determine if an internet-based intervention would be of interest to this group, the relationship between level of internet use and interest in using the internet for health was also examined.

## Methods

### Design and setting

A cross-sectional survey, which composed of existing or adapted validated survey items [26, 27], was conducted at two non-government community service organisations (CSO) in two major cities in New South Wales, Australia, from October 2013 to July 2014. CSOs provide financial and accommodation emergency aid to people in crisis. Smoking rates are high amongst CSO clients (between 60 and 70%) who tend to be homeless, unemployed, living with a mental illness, or identify as being Aboriginal or Torres Strait Islander [28, 29].

Clients were informed by CSO staff that a health survey was being conducted in the organisation. A Research Assistant approached clients to participate in the research, assessed eligibility and provided an information statement. The survey was self-completed using a touchscreen computer and survey completion was regarded as consent. Participants received a $10 grocery card gift voucher as reimbursement for completing the survey. Ethics approval for this study has been obtained from the University of Newcastle Human Research Ethics Committee (HREC-2010-1002).

### Participants

Clients of the CSOs were eligible if they were: (1) aged 18 years or older; (2) not under the influence of alcohol or other drugs or too distressed at time of recruitment; and (3) current daily or occasional smokers. Self-reported smoking status was assessed using the following two items (1) *“Do you currently smoke tobacco products?”* (response options: yes, daily; yes, at least once a week; yes, but less often than once a week; no, not at all) and (2) *“Have you smoked at least 100 cigarettes or a similar amount of smoking in your life?”* (response options: yes; no; not sure). Current smokers were defined as self-reported daily or occasional smokers who had smoked at least 100 cigarettes in their lifetime.

### Measures

#### Characteristics

##### Participant sociodemographics

Age, gender, Aboriginal and/or Torres Strait Islander (Indigenous Australian) status, education, marital status, housing status, weekly net income, and source of income were assessed.

##### Smoking status and smoking related variables

Nicotine dependence was assessed using the two-item Heaviness of Smoking Index (HSI) with higher scores indicating higher levels of nicotine dependence [26]. Quit intentions were measured by asking *“What are your intentions regarding quitting? Do you plan to;”* (response options: quit in the next 30 days; quit in the next 6 months; quit, but not in the next 6 months; never quit; don’t know). Self-efficacy for quitting was determined using the following: *“If you decided to give up smoking completely in the next 6* *months, how sure are you that you would succeed?”* (response options: not at all sure, slightly sure, moderately sure, very sure, extremely sure) [27].

Cannabis use was determined by asking *“During the past month how often did you use cannabis?”* (response options: 6–7 days each week; 4–5 days each week; 2–3 days each week; 1 day each week; 1 day each fortnight; once in the last month; not at all in the last month). This variable was dichotomised as a yes (in the last month) vs no (not at all) for regression modelling.

#### Outcome variables

##### Internet access

Internet access in the last 12 months was determined by asking *“In the last 12* *months, how often have you accessed the internet?”* (response options: every day; about once a week; less than once a week; not at all); these were collapsed into ‘at least weekly’ yes vs no for regression modelling.

##### Devices used to access the internet

Participants were asked to indicate yes or no to the question *“In the last 12* *months did you access the internet through any of the following?”* for the following devices: computer (desktop or laptop); smart phone; tablet; device not owned by you; other.

##### Using the internet to improve your health

Respondents were asked to indicate their interest in using the internet to improve their health by responding to the following *“Would you use the internet to help you improve your health?”* (response options: yes; no).

### Analysis

All data were stored on secure servers at the University of Newcastle and SAS v9.4 (SAS Institute Inc., Cary, NC, USA.) was used for analysis.

Descriptive statistics of participant sociodemographic characteristics are presented as numbers and percentages for categorical variables and means, medians, standard deviation (SD), minimum, and maximum for continuous variables. Logistic regressions were used to examine the associations between age, gender, heaviness of smoking, Indigenous Australian status, cannabis use, income, education, housing, and quit intentions with at least weekly internet use, device used for accessing the internet, and use of the internet for health improvement. Variables included in each regression model were selected a priori based on literature and clinical knowledge or were factors of interest as outlined in the introduction, with differences found by education, income, ethnicity, age, gender, smoking related variables, and marital status. Indigenous Australian status was split into two groups: Indigenous (Aboriginal and/or Torres Strait Islander) vs non-Indigenous individuals. Marital status was also categories into two groups: individuals who were married/de facto/living with partner vs separated/divorced/never married or single/widowed. Collinearity of variables in adjusted models were checked using variance inflation factors (VIFs) and by examining crude and adjusted estimates; no variables were found to be collinear, with all VIFs less than two, and adjusted estimates similar in effect size and direction to crude estimates. Crude and adjusted odds ratios with 95% confidence intervals and *p* values were calculated for variables in the models. Significance was determined at *p* < 0.05.

## Results

Of the 606 clients attending the two centres during the study period, 478 (78%) clients were eligible to take part and invited to see the Research Assistant for more information about the study. Reasons for ineligibility included being a non-smoker (n = 96), being under the influence of alcohol or other drugs (n = 5), distress (n = 3), and being aged under 18 years (n = 5). Of eligible clients, 369 (77%) participants consented and gave complete survey data.

*Participant sociodemographic characteristic.* Table 1 contains a summary of participant sociodemographic information. More participants were female (59%; n = 219), and average age was 40 years (SD = 11). Participants self-reporting as Aboriginal and/or Torres Strait Islander made up 21% (n = 78) of the sample, compared to 2.9% of the population in New South Wales [30]. Most of the sample reported low income with 71% (n = 261) reporting income well below the Australian single-person ‘poverty line’ of $413 per week [31] and 91% (n = 337) dependent on government benefits as their main source of income.

**Table 1:** Sociodemographic of the sample

Characteristic	Response option	Total (n = 369) n (%)
Age	Mean (SD)	40 (11)
	Median (min, max)	38 (19, 88)
Gender	Male	150 (41%)
Housing status	Own house	11 (3.0%)
	Rental house	142 (38%)
	With family or friends/hotel, motel/no home, street living	53 (14%)
	Supported accommodation/government housing	152 (41%)
	Other	11 (3.0%)
Indigenous Australian status	Aboriginal and/or Torres Strait Islander	78 (21%)
Marital status	Married/defacto/living with partner	72 (20%)
Highest level of education	Primary school	61 (17%)
	Secondary or less	236 (64%)
	Tertiary qualifications	72 (20%)
Weekly income amount (net)	Less than $200 per week	100 (29%)
	Between $201 and $400 per week	161 (47%)
	More than $400 per week	78 (23%)
Source of income	Paid employment (either full or part time)	18 (4.9%)
	Government pension or benefit	337 (91%)
	Other	14 (3.8%)

*SD* standard deviation

*Smoking characteristics* The majority of participants were current daily smokers, with a medium level of nicotine addiction (characterised as a Heaviness of Smoking Index score of 3–4). There was uncertainty around quit intentions, motivation and self-efficacy, however the majority (88%) were a bit or very interested in quitting (Table 2).

**Table 2:** Smoking characteristics

Characteristic	Response option	Total (n = 369) n (%)
Do you currently smoke tobacco products?	Yes, daily	338 (92%)
	Yes, at least once a week	22 (6.0%)
	Yes, but less often than once a week	9 (2.4%)
HSI	Mean (SD)	3 (2)
	Median (min, max)	3 (0, 6)
Number of cigarettes smoked per day	Mean (SD)	15.9 (10.0)
	Median (min, max)	15.0 (1.0, 50.0)
Lifetime quit attempts	Yes	303 (82%)
Quit attempt > 6 months	No quit attempt	66 (18%)
	< 6 months	238 (66%)
	> 6 months	59 (16%)
	Missing	6
Intentions regarding quitting	Don’t know	132 (36%)
	Never quit	19 (5.1%)
	Quit but not in the next 6 months	52 (14%)
	Quit in the next 6 months	112 (30%)
	Quit in the next 30 days	54 (15%)
Motivation to quit	Low motivation	74 (20%)
	Moderate motivation	174 (48%)
	High motivation	117 (32%)
	Missing	4
Interest in quitting	I am not interested in quitting smoking	43 (12%)
	I am a bit interested in quitting smoking	157 (43%)
	I am very interested in quitting smoking	169 (46%)
Self-efficacy	Not at all sure	128 (35%)
	Slightly sure	74 (20%)
	Moderately sure	100 (27%)
	Very sure	53 (14%)
	Extremely sure	14 (3.8%)
Cannabis use, previous month	Yes	104 (28%)

*HSI* Heaviness of Smoking Index, *SD* standard deviation

### Rates of internet use and type of technology used to access the internet

Fifty-eight percent of participants indicated that they used the internet at least weekly (n = 213). Access per device type ranged from 8% (other device), 32% (tablet), 65% (computer) to 75% (smart phone), with 58.8% using multiple device types (i.e. stating yes to more than one device). Approximately half (56%) of the respondents indicated that they would use the internet to improve their health.

### Association between participant characteristics and at least weekly internet use

Results from the logistic regression examining factors associated with at least weekly internet usage (Table 3) found that the odds of using the internet at least weekly internet was lower as age increased (per 1 year increase in age; OR = 0.94, 95% CI 0.92, 0.97, p < 0.001), and as HSI increased (per 1 unit increase; OR = 0.81, 95% CI 0.68, 0.97, p = 0.022) (Table 3). The odds of using the internet were higher if the participant had received a tertiary education (OR = 3.57, 95% CI 1.50, 8.54, p = 0.004) compared to a primary school education. Additionally, the odds of at least weekly internet use were higher for individuals earning $201–$400 per week compared less than $200 per week (OR = 2.74, 95% CI 1.52, 4.91, p < 0.001) and for individuals earning more than $400 per week compared to less than $200 per week (OR = 2.83, 95% CI 1.35, 5.95, p = 0.006).

**Table 3:** Characteristics related to reporting at least weekly internet use

Characteristic	At least weekly internet access^a^ n (%)	Crude^b^		Full^c^	
		Odds ratio (95% CI)	*p* value	(95% CI)	*p* value
Age^a^	58.1%	0.95 (0.93, 0.97)	*< 0.001*	0.94 (0.92, 0.97)	*<* *0.001*
Gender					
Male	71 (47%)	Ref		Ref	
Female	142 (65%)	2.05 (1.34, 3.14)	*<* *0.001*	1.64 (0.96, 2.81)	0.070
HSI	57.9%	0.80 (0.70, 0.92)	*0.001*	0.81 (0.68, 0.97)	*0.022*
Aboriginal and**/**or Torres Strait Islander			0.435		0.269
Non-Indigenous	171 (59%)	Ref		Ref	
Indigenous	42 (54%)	0.82 (0.50, 1.35)		0.7 (0.37, 1.32)	
Cannabis use			0.158		0.660
No	159 (60%)	Ref		Ref	
Yes	54 (52%)	0.72 (0.46, 1.14)		0.88 (0.50, 1.55)	
Education			*0.002*		*0.008*
Primary school	26 (43%)	Ref		Ref	
Secondary school	134 (57%)	1.77 (1.00, 3.12)	0.050	1.35 (0.68, 2.69)	0.388
Tertiary school	53 (74%)	3.76 (1.81, 7.79)	*<* *0.001*	3.57 (1.50, 8.54)	*0.004*
Housing			0.384		0.163
Own house	5 (45%	Ref		Ref	
Rental	82 (58%)	1.64 (0.48, 5.63)	0.432	2.40 (0.44, 13.04)	0.312
With family or friends/hotel, motel/no home, street living	28 (53%)	1.34 (0.36, 4.95)	0.657	1.87 (0.31, 11.31)	0.495
Supported accommodation/government housing	94 (62%)	1.94 (0.57, 6.66)	0.290	4.07 (0.73, 22.73)	0.109
Other	4 (36%)	0.69 (0.12, 3.78)	0.665	1.84 (0.20, 16.60)	0.585
Income amount			*<* *0.001*		*0.001*
≤ $200/week	42 (42%)	Ref		Ref	
$201–400/week	101 (63%)	2.32 (1.40, 3.87)	*0.001*	2.74 (1.52, 4.91)	*<* *0.001*
> $400/week	57 (73%)	3.75 (1.98, 7.10)	*<* *0.001*	2.83 (1.35, 5.95)	*0.006*
Cessation intention			0.723		0.071
Quit not next 6 months/Never quit	44 (62%)	Ref		Ref	
Quit within next 6 months	94 (57%)	0.80 (0.45, 1.42)	0.445	0.43 (0.21, 0.88)	0.022
Don’t know	75 (57%)	0.81 (0.45, 1.46)	0.477	0.59 (0.28, 1.22)	0.156
Marital status			0.907		0.659
Separated/divorced/never married or single/widowed	171 (58%)	Ref		Ref	
Married/defacto/living with partner	42 (58%)	1.03 (0.61, 1.74)		0.86 (0.44, 1.68)	
Self-efficacy^a^	57.8%	1.19 (1.00, 1.41)	0.056	1.12 (0.89, 1.41)	0.319

Italicised text indicates significance < 0.05

*HSI* Heaviness of Smoking Index

^a^ At least weekly internet use rate at the mean of the continuous variables, i.e. at the mean age, the rate of at least weekly internet use was 58.1%

^b^ Number of participants included in each crude model was 369 except for income amount (n = 339) and HSI (n = 366)

^c^ Full model (n = 336) included age, gender, HSI, Indigenous Australian status, cannabis use, education, housing, income amount, cessation intentions, marital status, and self-efficacy

### Relationship between participant characteristics with type of device used

Income was found to be related to using a computer to access the internet, with individuals earning $201–$400 per week reporting more than 2 times the odds of using a computer to access the internet than individuals earning less than $200 per week (OR = 2.35, 95% CI 1.19, 4.64, p = 0.014, Table 4).

**Table 4:** Significant characteristics related to device used to access the internet (non-significant associations not presented)

	Device use n (%)	Crude		Full^b^	
		Odds Ratio (95%)	*p* value	Odds Ratio (95%)	*p* value
*Computer*					
Income amount			*0.035*		*0.045*
≤ $200/week	31 (52.5%)	Ref		Ref	
$201–400/week	88 (72.1%)	2.34 (1.2, 4.5)	*0.010*	2.35 (1.19, 4.64)	*0.014*
> $400/week	43 (67.2%)	1.85 (0.89, 3.84)	0.099	1.95 (0.90, 4.25)	0.091
*Smartphone*					
Age^a^	74%	0.93 (0.90, 0.96)	*<* *0.001*	0.92 (0.89, 0.95)	**<** *0.001*
Cannabis			0.150		*0.011*
No	150 (76.9%)	Ref		Ref	
Yes	47 (68.1%)	0.64 (0.35, 1.17)		0.37 (0.18, 0.80)	
Income amount			*0.024*		*0.030*
≤ $200/week	50 (84.6%)	Ref		Ref	
$201–400/week	81 (66.4%)	0.36 (0.16, 0.79)	*0.012*	0.30 (0.12, 0.74)	*0.009*
> $400/week	50 (78.1%)	0.64 (0.26, 1.62)	0.349	0.47 (0.17, 1.34)	0.157
*Tablet use*					
Age^a^	29.8%	0.96 (0.94, 0.99)	*0.005*	0.96 (0.93, 0.99)	*0.008*
*Not my (other) device*					
Age^a^	38.8%	0.97 (0.94, 0.99)	*0.005*	0.97 (0.94, 1.00)	*0.021*

Italicised text indicates significance < 0.05

^a^ Rate at mean of continuous variables

^b^ Full model included age, gender, HSI, Indigenous Australian status, cannabis use, income amount, cessation intentions, marital status, and self-efficacy

Number of participants included in crude and full modelling was: computer crude n = 245 full model n = 243, smartphone crude age (n = 264) cannabis (n = 264) income amount (n = 245) full n = 243, tablet use crude n = 264 full n = 243, not my (other) device crude n = 264 full n = 243

Increasing age was found to have lower odds of using a smartphone (8% less likely per year older; OR 0.92, 95%CI 0.89, 0.95, p < 0.001), tablet (4% less likely per year older; OR 0.96, 95% CI 0.93, 0.99, p = 0.008), or other device (3% less likely per year older; 0.97, 95% CI 0.94, 1.00, p = 0.021) to access the internet in the last 12 months. Individuals earning $201–$400 per week were 70% less likely to report using a smartphone to access the internet (OR = 0.30, 95% CI 0.12, 0.74, p = 0.009), but were 2.3 times more likely to access the internet using a computer (OR = 2.35, 95% CI 1.19, 4.64, p = 0.014). Those participants who had used cannabis in the last month were 63% less likely (OR 0.37, 95% CI 0.18, 0.80, p = 0.011) to use a smartphone to access the internet in the last 12 months (Table 4).

### Using the internet for health and at least weekly internet access

Logistic regression analysis of the relationship between using the internet at least weekly and interest in using the internet for health was not found to be significant, even after adjusting for age, gender, heaviness of smoking, Indigenous Australian status, cannabis use, cessation intentions, income, marital status, and self-efficacy (model results not presented, OR 0.98, 95% CI 0.48, 2.01, p = 0.955).

## Discussion

This study describes the frequency with which people accessed the internet among a highly socially disadvantaged sample of Australian smokers. At least weekly internet access was found to be moderate (58%) for the total sample, however it was far below the national average of 86% [16]. The findings of this study suggests younger individuals, who are on the higher end of the low SES scale, and who have a lower level of nicotine addiction may therefore benefit most from an internet-based intervention aimed at socially disadvantaged individuals. Previous research has found that younger individuals who have a lower level of nicotine addiction are more likely to choose an internet-based intervention for smoking cessation when offered the choice between an internet only intervention or an internet/telephone combination intervention [32]. Interestingly, about half of the respondents indicated that they would be interested in using the internet for health though this was not found to be significantly related to greater frequency of internet use. Therefore, the development of an internet-based interventions could potentially assist in helping over half of a hard to reach group of socially disadvantaged individuals, however it may not be accessible to those who would be interested in using it (i.e. those who are interested in using the internet for health do not have at least weekly internet access). While developing and internet intervention could help to address the ‘reach’ of public health interventions, which, as discussed by Glasglow et al., is essential in refocusing the aims of healthcare to incorporate the needs of the less advantaged individuals, this too could add to the inequalities, with approximately 42% of individuals still potentially missing out [12, 13]. More research in this area is needed to determine which individuals precisely would be interest in using the internet for health and what platform they would prefer to facilitate access.

The odds of at least weekly internet access was found to be higher among younger individuals or among individuals who smoked at lower levels. These findings suggest that an internet-based smoking cessation intervention, such as the nationally available government website QuitNow [33], may be most accessible to younger individuals who smoke at lower rates. Therefore, older individuals, or those with higher levels of nicotine addiction may be the profile of individuals who are potentially missing out. This may suggest that even among socially disadvantaged groups, further disadvantage may exist for those who are older and/or smoke at heavier rates. A lower level of educational attainment, and a lower weekly income was also found to be linked to limited internet access. These differences within an already disadvantaged group of individuals cannot be overlooked, and may add to the health inequalities already seen, especially as older individuals who smoke more heavily may suffer from the impact of smoking related health conditions more immediately and severely then younger individuals who smoke at lower levels. For this reason, it is important to continue to develop novel interventions to continue to assist hard to reach populations.

Rates of internet access were comparable between Indigenous and non-Indigenous Australian respondents. These results indicate that an internet-based interventions may be appropriate especially given previously found support for internet-based interventions among Indigenous Australian communities [34, 35]. This is interesting as younger female Aboriginal Australians have been identified as using the internet more, with the role they play identified as being important as facilitating internet access for families [22]. The potential for an internet-based intervention which is culturally sensitive may therefore have a huge benefit given the greater than average percent of people who identify as Indigenous Australian in this socially disadvantaged population. This could also address some aspects of reach noted by Glasgow et al. as being important in the healthcare system [13].

The finding that at least half of the participants reported an interest in using the internet for health is promising as interest may indicate program uptake, usage, and retention. Retention to internet-based interventions in real-world conditions is often low, with retention rates found to average 50% (range 1–93%) [36]. Further, as access to the internet inevitability increases with technological advances, rates of interest in using the internet for health may too increase. Therefore, it is important to have effective internet-based interventions in place to capitalise on growing usage and interest. The inclusion of design techniques recommended to increase and maintain engagement and retention to program completion such as: tailoring; intervention design (i.e. web design, inclusion of behaviour change principals, theoretical bases etc.); use of graphics and videos; and prompts and reminders may also be beneficial. This is supported by Brown et al. who found that a more intensive intervention, one which was based in theory, included evidenced based behaviour change techniques, and principals from user testing with current tobacco smokers, was more effective when compared to information only for low SES individuals [37].

Finally, using a computer to access the internet was greater for individuals who reported earning ≥ $201 per week compared to individuals earning ≤ $200 per week. Conversely, using a smartphone to access the internet decreased as individuals reported higher income. This may suggest that there are differences in technologies used to access the internet among these groups. Therefore, designing an internet-based intervention that has both desktop (computer) and mobile device (smartphone, tablet) functionality is another important aspect to consider in program design, one which could also capitalise on increases in connectivity and individual interest, and one which could increase reach to a larger proportion of disadvantaged individuals.

## Limitations

A limitation of this study is that all results were based on self-report which can be biased by social desirability or errors of recall. The small number of measures included in the survey may have also been a limiting factor. Further, this research was limited to two NSW CSO services in an urban setting and therefore the results of this study may not generalise to other socially disadvantaged groups. However, this study has a robust sample of 369 highly socially disadvantaged smokers, who are often referred to as hard-to-reach, [38] with high rates of homelessness, poverty, and Indigenous Australian status. For this reason it is a novel representation of this population. Further, this data was collected in 2013–2014, meaning that these results may have changed. However, given that there remains a dearth of research on Internet access for disadvantaged groups, and that rates of access in Australia as a nation has not increased dramatically, there is a high probability that these findings are still the case for the majority of socially disadvantaged individuals.

## Conclusions

Internet access in a sample of socially disadvantaged smokers is lower (58%) than national average of 86% [16], suggesting that internet support will be potentially beneficial to more than half of a hard to reach group of at risk individuals. Internet-based interventions may have the potential to assist some of the most disadvantaged individuals, specifically younger smokers with a low level of nicotine addiction who may find it hard to quit, and therefore should be tailored to suit this target population. That the majority (89%) of participant indicated an interest in using the internet for health is a promising finding suggesting that this form of care would have support, especially if deployed on a desktop (computer) and mobile device (smartphone, tablet) to have the greatest reach. However, exacerbation of health inequalities may be seen among socially disadvantaged people who smoke tobacco, with these findings suggesting that heavier older smokers may not have as much access and therefore may not benefit from an internet-based intervention. For this reason, it is important to continue to establish novel approaches to providing increased care to these individuals.

## Data Availability

The datasets used and/or analysed during the current study are available from the corresponding author on reasonable request.

## Abbreviations

OR: odds ratio
SES: socioeconomic status
CSO: community service organisations
HREC: Human Research Ethics Committee
HIS: Heaviness of Smoking Index
SD: standard deviation
IQR: interquartile range
VIFs: variance inflation factors
NHMRC: National Health and Medical Research Council
